# Correction: Unexpected formation of 1,2- and 1,4-bismethoxyl Sc_3_N@*I*_h_-C_80_ derivatives *via* regioselective anion addition: an unambiguous structural identification and mechanism study

**DOI:** 10.1039/d1sc90116h

**Published:** 2021-05-26

**Authors:** Yajing Hu, Yang-Rong Yao, Xuechen Liu, Ao Yu, Xiaoming Xie, Laura Abella, Antonio Rodríguez-Fortea, Josep M. Poblet, Takeshi Akasaka, Ping Peng, Qianyan Zhang, Su-Yuan Xie, Fang-Fang Li, Xing Lu

**Affiliations:** State Key Laboratory of Material Processing and Die & Mould Technology, School of Materials Science and Engineering, Huazhong University of Science and Technology Wuhan Hubei 430074 China ffli@hust.edu.cn lux@hust.edu.cn; State Key Laboratory for Physical Chemistry of Solid Surfaces, Department of Chemistry, College of Chemistry and Chemical Engineering, Xiamen University Xiamen 361005 China xmuzhangqy@xmu.edu.cn; Departament de Química Física i Inorgànica, Universitat Rovira i Virgili Marcel·lí Domingo 1 43007 Tarragona Spain

## Abstract

Correction for ‘Unexpected formation of 1,2- and 1,4-bismethoxyl Sc_3_N@*I*_h_-C_80_ derivatives *via* regioselective anion addition: an unambiguous structural identification and mechanism study’ by Yajing Hu *et al.*, *Chem. Sci.*, 2021, DOI: 10.1039/d1sc01178b.

The authors regret a mistake in Fig. 3, showing the ^13^C NMR spectrum of product **1**. In the ^13^C NMR spectrum of **1**, the peaks corresponding to the sp^3^ carbons of the fullerene cage and the methoxy groups were wrongly identified. The correct version of Fig. 3 is shown below.

**Fig. 3:**
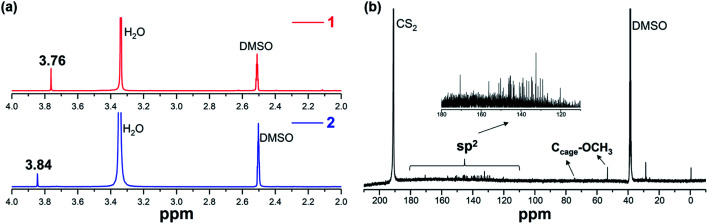
(a) ^1^H NMR spectra of **1** and **2** and (b) ^13^C NMR spectrum of **1** recorded in CS_2_ with DMSO-*d*_*6*_ as the external lock solvent.

The description of the ^13^C NMR spectrum in the Results and discussion section should therefore read: Resonance for the two sp^3^*I*_h_-C_80_ cage-carbons bonded to the OCH_3_ groups appears at 74.49 ppm, while the peak for the two sp^3^ OCH_3_ carbons appears at 53.76 ppm.

The description of the ^13^C NMR results in the Experimental section should therefore read: 74.49 (2C, sp^3^, C_cage_–OCH_3_), 53.76 ppm (2C, sp^3^, –OCH_3_).

These corrections do not influence any conclusions of the original paper.

The Royal Society of Chemistry apologises for these errors and any consequent inconvenience to authors and readers.

